# Breakthrough Invasive Fungal Infections in Allogeneic Hematopoietic Stem Cell Transplantation

**DOI:** 10.3390/jof7050347

**Published:** 2021-04-28

**Authors:** Carmine Liberatore, Francesca Farina, Raffaella Greco, Fabio Giglio, Daniela Clerici, Chiara Oltolini, Maria Teresa Lupo Stanghellini, Federica Barzaghi, Paolo Vezzulli, Elena Orsenigo, Consuelo Corti, Fabio Ciceri, Jacopo Peccatori

**Affiliations:** 1 Hematology and Bone Marrow Transplant Unit, IRCCS San Raffaele Scientific Institute, Via Olgettina 60, 20132 Milan, Italy; liberatore.carmine@hsr.it (C.L.); farina.francesca@hsr.it (F.F.); greco.raffaella@hsr.it (R.G.); giglio.fabio@hsr.it (F.G.); clerici.daniela@hsr.it (D.C.); lupostanghellini.mariateresa@hsr.it (M.T.L.S.); corti.consuelo@hsr.it (C.C.); peccatori.jacopo@hsr.it (J.P.); 2 Faculty of Medicine and Surgery, Vita-Salute San Raffaele University, IRCCS San Raffaele Scientific Institute, 20132 Milan, Italy; 3 Clinic of Infectious Diseases, Division of Immunology, Transplantation and Infectious Diseases, IRCCS San Raffaele Scientific Institute, 20132 Milan, Italy; oltolini.chiara@hsr.it; 4 Pediatric Immunohematology and Bone Marrow Transplantation Unit, IRCCS San Raffaele Scientific Institute, 20132 Milan, Italy; barzaghi.federica@hsr.it; 5 San Raffaele Telethon Institute for Gene Therapy (SR-Tiget), IRCCS San Raffaele Scientific Institute, 20132 Milan, Italy; 6 Neuroradiology Department, IRCCS San Raffaele Scientific Institute, 20132 Milan, Italy; vezzulli.paolo@hsr.it; 7 Department of General and Emergency Surgery, IRCCS San Raffaele Scientific Institute, 20132 Milan, Italy; orsenigo.elena@hsr.it

**Keywords:** invasive fungal infections, breakthrough invasive fungal infections, transplantation, chemotherapy, hematology, prophylaxis, antifungal therapy, new risks, diagnosis, resistance

## Abstract

Despite the recent introduction of mold-active antifungal prophylaxis (MAP), breakthrough invasive fungal infections (b-IFI) still represent a possible complication and a cause of morbidity and mortality in hematological patients and allogeneic hematopoietic stem-cell transplantation recipients (HSCT). Data on incidence and type of b-IFI are limited, although they are mainly caused by non-*fumigatus Aspergillus* and non-*Aspergillus* molds and seem to depend on specific antifungal prophylaxis and patients’ characteristics. Herein, we described the clinical presentation and management of two cases of rare b-IFI which recently occurred at our institution in patients undergoing HSCT and receiving MAP. The management of b-IFI is challenging due to the lack of data from prospective trials and high mortality rates. A thorough analysis of risk factors, ongoing antifungal prophylaxis, predisposing conditions and local epidemiology should drive the choice of antifungal treatments. Early broad-spectrum preemptive therapy with a lipid formulation of amphotericin-B, in combination with a different mold-active azole plus/minus terbinafine, is advisable. The therapy would cover against rare azole-susceptible and -resistant fungal strains, as well as atypical sites of infections. An aggressive diagnostic work-up is recommended for species identification and subsequent targeted therapy.

## 1. Introduction

In the era of mold-active antifungal prophylaxis (MAP), invasive fungal infections (IFI) still represent one of the major cause of morbidity and mortality in hematological patients and allogeneic hematopoietic stem-cell transplantation (HSCT) recipients. Following the decrease of invasive candidiasis due to fluconazole prophylaxis, an epidemiological shift occurred toward mold infections, mainly invasive aspergillosis (IA). Depending on risk factors, the 12-months IFI incidence approaches 8%, reaching 17% in haploidentical and cord blood transplantations [1,2]. Recently, ECIL-6 guidelines recommended primary MAP in high-risk HSCT recipients [3]. Nonetheless, MAP exposes the patient to development of breakthrough IFI (b-IFI), mainly by rare non-*Aspergillus* molds. To date, data on incidence and type of b-IFI are limited, although they seem to depend on specific prophylaxis and patients’ characteristics [4,5,6].

We described two cases of rare b-IFI which recently occurred at our institution in patients receiving MAP.

## 2. Case Descriptions, Methods and Results

Case 1: a 52-year-old woman diagnosed with acute myeloid leukemia (AML) was referred to our institution for HSCT. Four years before, she underwent HSCT from a matched unrelated donor (MUD), which was complicated by acute and chronic graft-versus-host-disease (GvHD). After a second haploidentical HSCT for disease reoccurrence, the FLT3-ITD mutated AML relapsed again. She received Gilteritinib for 6 months without MAP, obtaining complete remission with incomplete hematological recovery.

**Figure 1:**
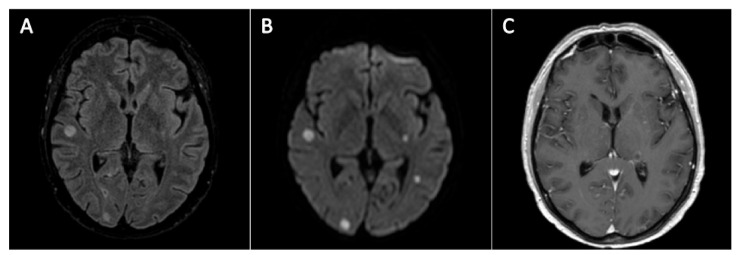
Magnetic resonance imaging of the brain for case 1. Findings were consistent with septic emboli. Pictures (**A**) and (**B**) show multifocal superficial and deep bilateral rounded lesions, some of which show nodular or ring-like enhancement after contrast injection (left thalamus in picture (**C**)). Lesions were localized at both sub- and sovra-tentorial level, with a miliary distribution, the largest ones involving the cerebellum and the brainstem with a maximum diameter of about 10 mm. In addition, phlogistic mucosal thickening of paranasal cavities was documented (not shown). (**A**): FLAIR sequence; (**B**): DWI sequence; (**C**): T1-post Gadolinium sequence.

At admission, the patient was in good clinical condition. Baseline evaluations were unremarkable, except for persistent severe neutropenia and cellulitis in left leg. Gilteritinib was stopped and cellulitis treated with daptomycin. Then, she received a reduced-intensity conditioning regimen followed by haploidentical HSCT. GvHD prophylaxis relied upon post-transplantation cyclophosphamide, mycophenolate-mofetil and sirolimus. Posaconazole was given as MAP from day -7 by therapeutic drug monitoring (TDM). On day +2 piperacillin/tazobactam was given for febrile neutropenia. Blood cultures and chest X-Ray were negative. A week later, fever reappeared, along with productive cough. Piperacillin/tazobactam was switched to meropenem, while posaconazole was continued. On day +11 she developed headache and diplopia, then dysarthria, drowsiness and ocular nystagmus. A brain magnetic resonance imaging scan showed multiple rounded lesions, diagnostic for septic embolism (Figure 1). Soon after, the patient developed hemorrhagic skin lesions and multi-organ failure. As galactomannan (GM) monitoring in serum was negative and preliminary results of both sputum and blood culture suggested a yeast-like fungus, posaconazole was switched to liposomal amphotericin-B (L-AMB) (5 mg/kg/day) and anidulafungin. Two days later, definitive results showed *Lomentospora prolificans* (Table 1); however, the same day, she went into a coma and died.

Case 2: a 34-year-old man diagnosed with immune-mediated thrombocytopenia (ITP) and primary immunodeficiency was referred to our institution for HSCT. ITP was refractory to several treatments, including steroids, immunoglobulins, rituximab, danazol, azathioprine and romiplostim. Investigations of pre-existent lymphopenia suggested a primary immunodeficiency. Although next-generation sequencing did not identify any pathogenetic variants, HSCT was indicated based on clinical phenotype.

At admission, the patient was in good clinical condition. He received a myeloablative conditioning regimen followed by HSCT from MUD. GvHD prophylaxis relied upon ATG, cyclosporine-A and methotrexate. Voriconazole was given as MAP from day −7 and by TDM. Complications were febrile neutropenia and severe mucositis, treated with piperacillin/tazobactam, then meropenem and tigecycline for ESBL-producer *Klebsiella pneumoniae* blood-stream infection. Neutrophils engraftment was achieved on day +15. Two weeks later the patient presented acute peritonitis. Abdomen CT scan showed ischemic jejunal perforation and he underwent emergency open surgery with intestinal resection and anastomosis. Considering critical condition, MAP was modified in favor of intravenous isavuconazole to contain toxicities. GM monitoring in serum was negative. Histopathological examination of jejunum revealed tissue necrosis and angioinvasion by fungal hyphae consistent with mucormycosis. Further foci of IFI were excluded and a combinational therapy adding L-AMB 5 mg/kg/day to isavuconazole was preferred while waiting for microbiological results. Subsequently, the patient presented cutaneous-subcutaneous necrosis around the surgical site, requiring multiple debridements and vacuum-assisted closure therapy. Histopathological examination of soft tissue confirmed mucormycosis and culture of tissue biopsy detected *Rhizopus microsporus* (Table 1). Fortunately, the patient had a slow but continuous improvement. L-AMB was administered for 45 days while oral isavuconazole was continued for a further 8 months during immunosuppression therapy (IST). At last follow-up (18 months after HSCT), he persisted in good clinical condition.

## 3. Discussion

The management of b-IFI during MAP is challenging, as no standardized prospective data are available to guide therapeutic decisions. Consensus statements suggest aggressive diagnostic work-up, multidisciplinary management and early broad-spectrum preemptive therapy with L-AMB, with or without a different mold-active azole, thus covering against azole-susceptible and -resistant fungal strains [7,8].

In patients receiving posaconazole, prospective trials reported b-IFI up to 3.7%: mainly IA and a few cases of lomentosporiosis [9,10]. Retrospective studies also reported incidence of b-IFI between 0–10.9%: *A. fumigatus* was the most common, followed by non-*fumigatus* IA, mucormycosis, fusariosis and, rarely, candidiasis [4,6,7]. *Lomentospora prolificans*, previously termed as *Scedosporium prolificans*, is a rare opportunist mainly affecting immunocompromised and hematological patients [11]. Lomentosporiosis accounts for 25% of all non-IA in solid organ and HSCT recipients [12]. Clinical manifestations range from localized to severe invasive mycoses. Risk factors for dissemination include: underlying disease (mainly acute leukemia), type of transplantation/IST, duration and degree of neutropenia, antifungal prophylaxis and environmental exposure [11,13]. Disseminated disease occurs more often in HSCT recipients, mainly in the pre-engraftment period, and frequently presents as fungemia, with a high percentage of isolation from blood cultures. It is characterized by mortality rates >80% due to intrinsically pan-antifungal resistance [11,13,14]. Results of our susceptibility test were consistent with those reported in literature. In a recent review of 41 cases of lomentosporiosis, combinational therapy with voriconazole plus terbinafine resulted in increased survival [15]. The ECCM-ASM guidelines actually recommend a first-line voriconazole-based combinational therapy, particularly with terbinafine, alongside surgical debridement and recovery of immunosuppression [8,16]. The novel compounds E1210/APX001 and F901318 showed preliminary in vitro activity, and in vivo clinical trials are ongoing [17,18]. In case 1, clinical presentation was suggestive for b-IFI and the patient had multiple risk factors, e.g., advanced stage heavily pretreated AML, long-lasting severe neutropenia, previous HSCT and IST for acute and chronic GvHD. Before the confirmation of lomentosporiosis, considering epidemiology, posaconazole prophylaxis, GM negativity and initial suggestion for yeast-like fungi, we chose a combinational therapy with L-AMB and anidulafungin. Although often detectable in aspergillosis, GM is typically negative in cases of mucormycosis, lomentosporiosis and scedosporiosis, because it does not constitute a component of the cell wall of these fungi. The role of GM monitoring in the diagnostic work-up of patients receiving MAP is controversial, due to lower sensitivity and specificity [19]. However, these findings were mainly confirmed by studies with Posaconazole as MAP, rather than all studies [20]. Therefore, GM monitoring is still routinely performed in many centers when a b-IFI is clinically suspected in the setting of high-risk hematological patients [7,21]. Unfortunately, in our experience, when definitive microbiological results became available, the rapid clinical worsening did not allow administration of an adequate antifungal therapy.

In case 2, suspicion of b-IFI was initially less consistent, as clinical scenarios could lead to other pathogens and noninfectious complications. Nonetheless, the patient had multiple risk factors for b-IFI, including prolonged steroid therapy, primary immunodeficiency, myeloablative conditioning, ATG administration and neutropenia post-HSCT. Moreover, voriconazole prophylaxis—as well as GM negativity—favored diagnosis of non-IA. Actually, b-IFI following voriconazole prophylaxis has been reported in up to 7.9% of cases, primarily mucormycosis, due to the lack of in vitro activity against *Mucorales* [4]. Mucormycosis, including *Rhizopus microsporus*, mainly affects immunocompromised hosts and, after IA, represents the most frequent IFI in hematological patients and HSCT recipients [22,23,24,25]. Mucormycoses are characterized by rapid local spread, angioinvasion and tissue necrosis. GM tests are typically negative. Most common presentations are rhino-orbito-cerebral, cutaneous, pulmonary and disseminated, the latter two being more frequent among neutropenic patients with acute leukemia and HSCT recipients. The latter two are also associated with high mortality rates (20–100%) [22,23,25,26]. The ESCMID-ECCM and ECIL-6 guidelines recommend surgery and first-line treatment with L-AMB at least 5 mg/kg/day. Posaconazole is an alternative in first-line and salvage settings, as well as combinational therapy of L-AMB with posaconazole or echinocandines [27,28]. A recent single-center trial testing first-line isavuconazole in mucormycoses reported similar outcomes compared to L-AMB [29]. The most recent ECMM guidelines still recommend first-line L-AMB, with intravenous isavuconazole or posaconazole in patients with renal impairment [30]. In case 2, as the pathologist suspected mucormycosis and considering MAP with voriconazole, disseminated infection, patient’s risk factors and unavailability of intravenous posaconazole, a combinational therapy with L-AMB and intravenous isavuconazole was chosen. In our experience, surgical debridement and combinational therapy proved effective. Clinical efficacy and susceptibility tests favored isavuconazole as a continuing oral treatment during IST.

The limitations of this case study include the retrospective nature, selected population and lack of generalizability.

## 4. Conclusions

This report described a real-life and patient-specific approach to diagnosis and management of b-IFI, providing data on species identification and susceptibility testing. b-IFI should still be considered a possible complication in HSCT recipients receiving MAP. Due to high mortality rates, early broad-spectrum preemptive therapy is advisable against even rare species and atypical sites of infections. A thorough analysis of risk factors and predisposing conditions should drive the choice of individualized antifungal treatments. Efforts are strongly recommended for species identification and susceptibility testing in order to establish a targeted therapy. As new transplantation strategies are implemented and the use of MAP is broadened, prospective studies are needed to better understand the epidemiology, clinical manifestations and outcomes of b-IFI.

## Figures and Tables

**Table 1:** In vitro antifungal susceptibility tests.

	Clinical Case 1	Clinical Case 2
**Species**	*Lomentospora prolificans*	*Rhizopus microsporus*
**Sample**	Peripheral blood	Soft tissue biopsy
**Drugs**	**MIC**	**MIC**
L-AMB	>32 mg/L	0.5 mg/L
Itraconazole	>32 mg/L	1.5 mg/L
Voriconazole	>32 mg/L	-
Posaconazole	>32 mg/L	0.19 mg/L
Isavuconazole	>32 mg/L	0.125 mg/L

Table 1 shows in vitro antifungal susceptibility tests of fungal strains isolated from patients (cases 1 and 2). Minimal inhibitory concentration was determined by E-test. Lacking validated MIC breakpoints for any of the drugs against Lomentospora and Mucor fungal genera, interpretation and determination of susceptibility categories was not possible. L-AMB: liposomal Amphotericin B.

## Data Availability

Not applicable.
